# Evaluation of ^99m^Tc-HYNIC-βAla-Bombesin_(7-14)_ as an agent for pancreas tumor detection in mice

**DOI:** 10.1590/1414-431X20154506

**Published:** 2015-05-19

**Authors:** F.N. Carlesso, L.L. Fuscaldi, R.S. Araújo, C.S. Teixeira, M.C. Oliveira, S.O.A. Fernandes, G.D. Cassali, D.C. Reis, A.L.B. Barros, V.N. Cardoso

**Affiliations:** 1 Faculdade de Farmácia, Universidade Federal de Minas Gerais, Belo Horizonte, MG, Brasil; 2 Instituto de Ciências Biológicas, Universidade Federal de Minas Gerais, Belo Horizonte, MG, Brasil

**Keywords:** ^99m^Tc-HYNIC-βAla-Bombesin, Capan-1, Scintigraphic images, Pancreas tumor, Biodistribution

## Abstract

Pancreatic adenocarcinoma is important in oncology because of its high mortality rate. Deaths may be avoided if an early diagnosis could be achieved. Several types of tumors overexpress gastrin-releasing peptide receptors (GRPr), including pancreatic cancer cells. Thus, a radiolabeled peptide derivative of gastrin-releasing peptide (GRP) may be useful as a specific imaging probe. The purpose of the present study was to evaluate the feasibility of using^99m^Tc-HYNIC-βAla-Bombesin_(7-14)_as an imaging probe for Capan-1 pancreatic adenocarcinoma. Xenographic pancreatic tumor was developed in nude mice and characterized by histopathological analysis. Biodistribution studies and scintigraphic images were carried out in tumor-bearing nude mice. The two methods showed higher uptake by pancreatic tumor when compared to muscle (used as control), and the tumor-to-muscle ratio indicated that^99m^Tc-HYNIC-βAla-Bombesin_(7-14)_uptake was four-fold higher in tumor cells than in other tissues. Scintigraphic images also showed a clear signal at the tumor site. The present data indicate that^99m^Tc-HYNIC-βAla-Bombesin_(7-14)_may be useful for the detection of pancreatic adenocarcinoma.

## Introduction

Cancer is one of the main causes of death worldwide. In 2012, about 14.1 million new cases of the disease were registered, resulting in approximately 8.2 million deaths. Pancreatic adenocarcinoma has a high mortality rate. Global data showed 337,872 new cases of this disease in 2012, followed by 330,372 deaths, which represent approximately 98% of the cases (1). Deaths could be delayed if an early diagnosis could be achieved.

Cancer cells are characterized by the pathological upregulation of several physiological processes, including the overexpression of a variety of peptide receptors in cancer cell membranes (2). This upregulation enables the use of radiolabeled peptides for the differentiation between tumor and normal tissues by molecular imaging. For example, pancreas, prostate, lung, colon and breast cancer cells present an increased expression of gastrin-releasing peptide receptors (GRPr). Thus, a radiolabeled peptide derivative of gastrin-releasing peptide (GRP) may be used as a specific imaging probe for these types of tumors (3-5).

The neuropeptide bombesin consists of 14 amino acids and is analogous to GRP, differing in only 1 of 10 carboxy-terminal residues. Bombesin has been evaluated as a radiotracer for GRPr-overexpressing tumors (6-8). A truncated sequence of bombesin containing the eight carboxy-terminal residues (Bombesin_(7-14)_) necessary to retain its affinity for GRPr has been employed in order to identify tumors. Coincidentally, the removal of the six nitrogen-terminal residues also increases its stability (9,10).

Technetium-99m (^99m^Tc) is the most widely used radionuclide in nuclear medicine since it presents suitable characteristics for a radioisotope, including a physical half-life of 6.02 h and gamma emission of low energy (140 keV) (11,12). For using ^99m^Tc for radiolabeling Bombesin_(7-14)_, which does not have a disulfide bond, 2-hydrazinonicotinamide (HYNIC) is a good chelating agent because its carboxylic acid group reacts with the nitrogen-terminal residue of the peptide. This addition does not affect Bombesin_(7-14)_ binding to GRPr, since it occurs near the carboxy-terminal residue. Moreover, a spacer amino acid, such as beta-alanine (βAla), can be added between HYNIC and Bombesin_(7-14)_ without compromising the peptide's interaction with its receptor. Finally, the presence of co-ligands such as tricine and ethylenediamine-N,N'-diacetic acid (EDDA) in the radiolabeling procedure of HYNIC-βAla-Bombesin_(7-14)_stabilizes the metal complex (13,14).

The Capan-1 cell line was isolated from a liver metastasis of a human pancreatic adenocarcinoma. This tumor cell line is of ductal origin and exhibits characteristics of a well-differentiated adenocarcinoma. In addition, it produces a well-defined tumor nodule after subcutaneous inoculation in nude mice, mimicking the tumor in humans (15). It has been reported that this ductal cell line expresses functional GRPr on its membrane surface (16-18). Therefore, a GRP analog such as a Bombesin derivative may be used to identify Capan-1 tumor tissues.

The objective of the present study was to develop pancreatic adenocarcinomas in nude mice using the Capan-1 cell line and to evaluate the feasibility of^99m^Tc-HYNIC-βAla-Bombesin_(7-14)_ as an imaging probe for Capan-1 pancreatic adenocarcinoma.

## Material and Methods

### Material

The peptide HYNIC-βAla-Bombesin_(7-14)_ was purchased from GL Biochem Ltd. (China). ^99m^Tc was obtained from a^99^Mo/^99m^Tc generator supplied by Instituto de Pesquisas Energéticas e Nucleares (IPEN; Brazil). Other reagents and solvents were acquired from Sigma-Aldrich (Brazil). The Capan-1 human cell line, Iscove's Modified Dulbecco's Medium (IMDM) and fetal bovine serum were purchased from American Type Culture Collection (ATCC; USA). Trypsin was acquired from Invitrogen (Brazil). Nude male BALB/c mice (18-20 g) were supplied by Fundação de Apoio e Fomento à Inovação Tecnológica, à Pesquisa e ao Ensino of IPEN (Brazil). Animals were kept under specific pathogen-free conditions, with*ad libitum* access to chow and water. The housing was temperature-controlled with filtered air and a light-dark cycle (12/12 h). The experiments were conducted according to animal-use principles approved by the local Ethics Committee on Animal Use of the Universidade Federal de Minas Gerais (CEUA/UFMG).

### Radiolabeling of HYNIC-βAla-Bombesin_(7-14)_

The radiolabeling procedure was performed according to a method published elsewhere (19). Briefly, 20 mg of tricine and 5 mg of EDDA were added to a sealed vial and dissolved with 0.5 mL of 0.9% NaCl (w/v). To this solution, 10 µg of HYNIC-βAla-Bombesin_(7-14)_ and 10 µL of a 1-mg/mL SnCl_2_·2H_2_O solution in 0.25 N HCl were added and vacuum was applied. The pH was adjusted to 7–8, and 74 MBq of Na^99m^TcO_4_ was added to the vial. Finally, the solution was heated in a water bath (100°C) for 15 min and cooled to room temperature. The specific activity of the final product was 8.5 MBq/nmol.

### Radiolabeling yield

Radiolabeling yields were determined by thin-layer chromatography (TLC) on silica gel strips (Merck^®^, Germany). Methyl ethyl ketone was used to determine the free technetium (^99m^TcO_4_^–^) and a solution of acetonitrile:water (1:1) was used to quantify hydrolyzed technetium (^99m^TcO_2_). Radioactivity was measured using an automatic gamma counter (Wizard, Finland).

### Capan-1 cell culture

Capan-1 cells were maintained at 37°C in a humidified atmosphere containing 5% CO_2_ and were continuously grown in IMDM supplemented with 10% (v/v) fetal bovine serum (FBS) (Sigma-Aldrich, USA), 100 IU/mL penicillin (Sigma-Aldrich), and 10 µg/mL streptomycin (Sigma-Aldrich). The cells were grown to confluence and then harvested by trypsinization. After centrifugation (5 min at 241*g*), cells were re-suspended in IMDM for development of the pancreas tumor in an animal model.

### Pancreas tumor animal model

An aliquot (100 µL) containing 5×10^6^ Capan-1 cells in IMDM was injected subcutaneously in the right upper flank of each nude mouse. Tumors were allowed to grow *in vivo* for 25 days after the inoculations, with a tumor diameter of no more than 10 mm being obtained at this time. Capan-1 tumor-bearing mice were then used for*ex vivo* biodistribution studies and scintigraphic images.

### *Ex vivo* biodistribution studies

Aliquots containing^99m^Tc-HYNIC-βAla-Bombesin_(7–14)_(7.4 MBq) were administered into the tail vein of Capan-1 tumor-bearing mice (n=5). After 4 h of radiolabeled peptide administration, the mice were anesthetized with a solution of 80 mg/kg ketamine and 15 mg/kg xylazine, and then euthanized. Organs and tissues of interest, such as spleen, heart, stomach, liver, small intestine, contralateral muscle, kidneys, blood, and tumor were removed and weighed. The associated radioactivity was determined with an automatic gamma counter (Wizard, Finland). Results are reported as percent of the injected dose per gram of tissue (% ID/g).

### Histopathological analysis

After performing *ex vivo* biodistribution studies, Capan-1 tumor tissues were fixed in formalin (10% w/v in phosphate-buffered saline, pH 7.4), and sections (4 µm) were prepared for light microscopy studies. All staining procedures were performed on paraffin-embedded sections mounted on glass slides. Histological section images were captured by a digital camera (Spot Insight Color, USA) adapted to an Olympus microscope (BX-40; Japan). SPOT^®^software (version 3.4.5) and CorelDRAW^®^ (version 11.633, USA) were used for image analysis.

### Scintigraphic images

^99m^Tc-HYNIC-βAla-Bombesin_(7–14)_(7.4 MBq) was administered into the tail vein of Capan-1 tumor-bearing mice. At 1 and 4 h after injection, the mice were anesthetized with 80 mg/kg ketamine and 15 mg/kg xylazine, and then placed in a prone position under a gamma camera (Mediso, Hungary) with a low-energy high-resolution collimator. Images were acquired using a 256×256×16 matrix size with a 20% energy window set at 140 keV for 10 min. We chose an early time (1 h) and a late time (4 h) in order to demonstrate the clearance of the radiolabeled peptide. Regions of interest (ROIs) were analyzed by scintigraphic images outlining the tumor (target). The ROIs were automatically copied to the contralateral muscle (non-target). The target/non-target ratios were calculated using the total ROI counts.

### Statistical analysis

Data are reported as means±SD. The means of the 2 groups of ROIs (target and non-target) were compared using Student's*t*-test. P values of less than 0.05 were considered to be significant. Data were analyzed using the Prism software (version 5.00, USA).

## Results and Discussion

### Radiolabeling of HYNIC-βAla-Bombesin_(7-14)_ and radiolabeling yield evaluation

HYNIC-βAla-Bombesin_(7-14)_ was successfully labeled with^99m^Tc, showing a radiolabeling yield of 97.72±0.73% (n=6). This yield is consistent with other works published by our group (19-21).

The presence of radiochemical impurities has proved to be a drawback in nuclear medicine, yielding images of poor quality. It is accepted that, to be injected, a radiopharmaceutical must present a radiolabeling yield higher than 90%. Therefore, the^99m^Tc-HYNIC-βAla-Bombesina_(7-14)_complex presented good chemical characteristics, since it showed high radiochemical purity (>95%).

### Histopathological analysis

**Figure 1:**
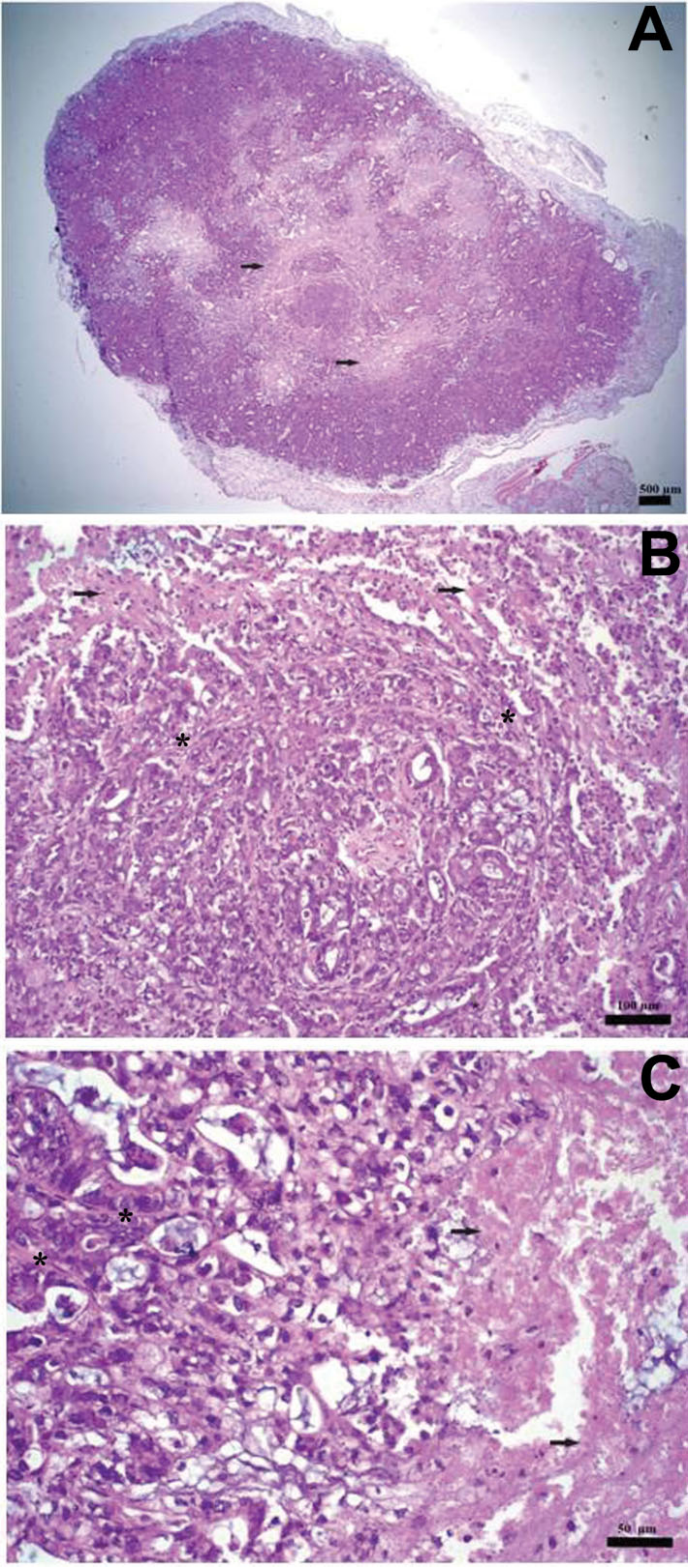
Pancreatic adenocarcinoma experimental model at 25 days after inoculation of the human ductal Capan-1 cell line into nude male BALB/c mice. Tumor histological section images, hematoxylin and eosin staining.*A*, 2×, scale bar: 500 µm;*B,* 20×, scale bar: 100 µm:*C*, 40×, scale bar: 50 µm. Arrows indicate areas of necrosis and the asterisks represent conjunctive stroma.

A xenographic pancreatic adenocarcinoma was developed by the subcutaneous inoculation of the human ductal Capan-1 cell line in the right upper flank of nude male BALB/c mice. In order to obtain a suitable tumor focus, cells were allowed to grow for about 25 days after their inoculation into the animals. Histopathological examination (Figure 1) revealed that tumor tissue presented a delicate conjunctive stroma and more necrosis in the central area. Histopathological analysis also showed evident mitotic activity and the presence of papillary projections and granular cells.

### Scintigraphic images and *ex vivo* biodistribution studies

**Figure 2:**
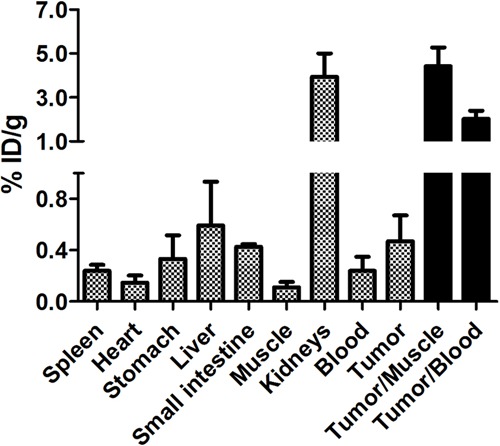
Biodistribution of^99m^Tc-HYNIC-Bombesin_(7-14)_ in Capan-1 tumor-bearing mice (% ID/g) after 4 h of intravenous injection of^99m^Tc-HYNIC-βAla-Bombesin_(7-14)_(7.4 MBq). Data are reported as means±SD. % ID/g: injected dose per gram of tissue.

**Figure 3:**
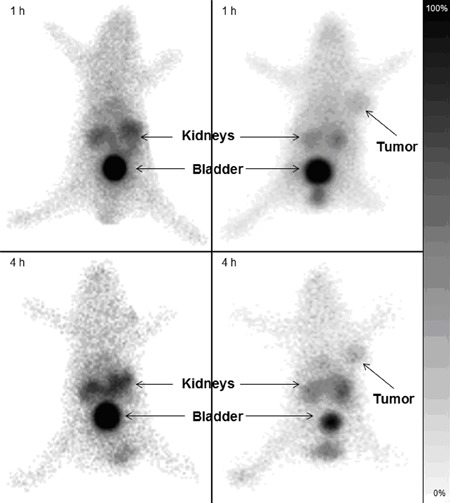
Scintigraphic images (256×256×16 matrix size) obtained at 1 and 4 h after^99m^Tc-HYNIC-βAla-Bombesin_(7-14)_intravenous injection (7.4 MBq) into healthy mice (*left*) and Capan-1 tumor-bearing mice (*right*) anesthetized with ketamine and xylazine. Arrows indicate tumor foci, kidneys and bladder.

*Ex vivo* biodistribution (Figure 2) showed that^99m^Tc-HYNIC-βAla-Bombesin_(7–14)_presented high kidney uptake. Scintigraphic images (Figure 3) corroborate the biodistribution results, showing high radioactivity accumulation in the abdominal region, which was mainly due to^99m^Tc-HYNIC-βAla-Bombesin_(7–14)_depuration, showing high levels of radioactivity in kidneys and bladder. These results indicate radiopeptide elimination by the urinary tract, which is consistent with the hydrophilic nature of the molecule (20-22). In addition, low levels of radioactivity were observed in stomach, liver, and spleen, corresponding to the low quantities of radiochemical impurities, such as^99m^TcO_4_^–^ and ^99m^TcO_2_.

**Figure 4:**
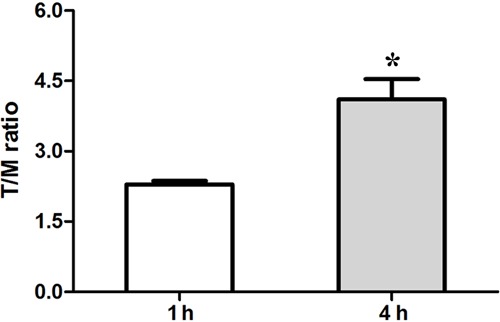
Quantitative analysis of tumor-to-muscle (T/M) ratio evaluated by scintigraphic images. Data are reported as means±SD. *P<0.05, 4 h compared to 1 h (unpaired*t*-test).

The xenographic pancreas tumor was successfully developed and identified 30 days after the subcutaneous inoculation of the Capan-1 human cell line into nude male BALB/c mice, as can be clearly seen by the scintigraphic images obtained 1 and 4 h after radiopeptide administration (Figure 3). Scintigraphic images showed higher uptake by the tumor (right thigh) when compared with the left thigh used as control. The tumor-to-muscle (T/M) ratio obtained by means of scintigraphic images (Figure 4) increased at 4 h (4.11±0.75) compared to the values detected at 1 h (2.29±0.13). This increase may be explained by a higher clearance of^99m^Tc-HYNIC-βAla-Bombesin_(7–14)_from blood and contralateral muscle compared to its elimination from the tumor focus. The visual analysis of scintigraphic images corroborated the quantitative data, since the tumor focus was more evident at 4 h than at 1 h after radiopeptide injection into Capan-1 tumor-bearing mice. This finding was due to an increase in the target/non-target ratio due to clearance of the radiopharmaceutical in the non-target organ.

*Ex vivo* biodistribution data showed that^99m^Tc-HYNIC-βAla-Bombesin_(7–14)_uptake by the tumor focus was 0.47±0.20% ID/g and demonstrated T/M and tumor-to-blood (T/B) of 4.42±0.85 and 2.02±0.36, respectively, at 4 h after radiopeptide administration. These results were similar to those obtained in a previous study by our group (19) in which an uptake of 0.39±0.03% ID/g was achieved in Ehrlich tumor-bearing mice, reaching T/M and tumor-to-blood (T/B) ratios of 4.78±0.81 and 3.53±0.53, respectively, at the same 4 h post injection. All target/non-target ratios from scintigraphic images and *ex vivo* biodistribution assay were higher than 1.5, which represents a 50% higher radiopeptide uptake by the target tissue than the by non-target tissues. Thus, the radiotracer is able to produce high quality images (23), as can be seen in the present study where^99m^Tc-HYNIC-βAla-Bombesin_(7–14)_was able to identify the Capan-1 focus at an earlier stage of tumor development.

Several published studies have reported *in vivo* profiles of^99m^Tc-HYNIC-βAla-Bombesin_(7–14)_similar to those obtained in the present study. The authors described radiopeptide accumulation in the kidneys, bladder, and tumor sites after its administration in murine breast (Ehrlich cells) (19), human breast (MDA-MB-231 cells) (24), prostate (PC3 and LNCaP cells) (21,22) and colon (HT-29 cells) (25) tumor-bearing mice. However, to the best of our knowledge, no previous study has evaluated the feasibility of^99m^Tc-HYNIC-βAla-Bombesin_(7–14)_to identify Capan-1 pancreatic adenocarcinoma.

The aggressiveness and high incidence of metastases associated with pancreatic adenocarcinoma represent a challenge for clinicians and researchers. A strategy to overcome some of these difficulties would be the early identification of the tumor. Genetic study of pancreatic cancer suggested an opportunity for the early detection of pancreatic neoplasia. However, this approach has some disadvantages since it is an invasive and an expensive methodology requiring a biopsy and expensive biochemical analysis, such as RT-PCR. Imaging methods for pancreatic tumors are innovative and rarely reported in the literature (26). Finally, the data presented here suggest that ^99m^Tc-HYNIC-Bombesin_(7–14)_could be used as a new potential agent to detect Capan-1 tumors by scintigraphic images at an early stage of tumor development. In addition, it could be used to monitor cancer therapy.
